# Low PARP-1 expression level is an indicator of poor prognosis in patients with stage II and III gastric cancer

**DOI:** 10.7150/jca.65145

**Published:** 2022-01-01

**Authors:** Song Ee Park, Hee Sung Kim, Eun-Jung Jung, Ja Hee Suh, Hyeyoung Min, Kyong-Choun Chi, Jong Won Kim, Joong-Min Park, In Gyu Hwang

**Affiliations:** 1 Department of Internal Medicine, Chung-Ang University College of Medicine, Seoul, Korea.; 2 Department of Pathology, Chung-Ang University College of Medicine, Seoul, Korea.; 3 Department of Pathology, National Medical Center, Seoul, Korea.; 4 College of Pharmacy, Chung-Ang University, Seoul, Korea.; 5 Department of Surgery, Chung-Ang University College of Medicine, Seoul, Korea.

**Keywords:** DNA damage response (DDR), Poly adenosine diphosphate-ribose polymerase 1 (PARP-1), Gastric cancer, Survival, Gastrectomy

## Abstract

**Purpose:** This study aimed to investigate the relationship between DNA damage response (DDR) related protein expression and clinical outcomes of patients with stage II and III gastric cancer undergoing gastrectomy.

**Materials and Methods:** From January 2005 to December 2017, 217 gastrectomized patients with stage II and III gastric cancer were analyzed for disease-free and overall survival (DFS and OS, respectively) based on their DDR expression status. We performed the immunohistochemical assessment of MLH1, MSH2, at-rich interaction domain 1 (ARID1A), poly adenosine diphosphate-ribose polymerase 1 (PARP-1), breast cancer susceptibility gene 1 (BRCA1), and ataxia-telangiectasia mutated (ATM) using formalin-fixed paraffin-embedded (FFPE) samples.

**Results**: Among the 217 patients studied, the most common DDR gene whose expression was suppressed was high PARP-1 (n = 120, 55.3%), followed by ATM (n = 62, 28.6%), ARID1A (n = 45, 20.7%), MLH1 (n = 33, 15.2%), BRCA1 (n = 25, 11.5%), and MSH2 (n = 9, 4.1%). The low-expression PARP-1 group exhibited a significantly shorter 5-year OS rate than the high-expression PARP-1 group (48.1% vs. 62.7%; HR 1.519, 95% CI = 1.011-2.283, P = 0.044). In the multivariate OS analysis, TNM stage (II vs. III) (HR = 5.172, P < 0.001), low PARP-1 expression (HR = 1.697, P = 0.013) and adjuvant chemotherapy (HR = 0.382, P < 0.001) were the only significant prognostic factors.

**Conclusions**: Low PARP-1 expression level could be an indicator of poor prognosis in gastrectomized patients with stage II and III gastric cancer.

## Introduction

Gastric cancer is reportedly the fifth most common cancer and the third leading cause of cancer-related deaths worldwide 1. At present, surgical resection and D1 or D2 gastrectomy are the main treatment approaches for stage II and III gastric cancer. However, even after curative resection, the 5-year survival rate is approximately 40-78% 2, 3. Adjuvant chemotherapy is the standard treatment associated with resectable gastric cancer therapy; it has been reported to improve patient survival 3. Adjuvant treatment reduces both distant and locoregional recurrences, although its related 5-year disease-free survival rate is poor (53-68%) 4. However, despite its potential relevance, no clinically relevant survival- and post-surgical relapse-related prognostic marker for gastric cancer has been identified yet.

When DNA damage occurs, DNA damage response (DDR) is activated within a cell cycle checkpoint 5. Defects in DDR could allow cell survival or the continuous growth of cancer cells 6. DDR-related proteins, such as MLH1, MSH2 7, AT-rich interaction domain 1A (ARID1A) 8, poly [ADP-ribose] polymerase 1 (PARP-1) 9, breast cancer susceptibility gene (BRCA1) 10, and ataxia-telangiectasia mutated protein (ATM) may allow cancer cells to evade physiological cell cycle checkpoints and facilitate cancer cell survival and proliferation.

DDR expression has been correlated with an improved response to cisplatin-based chemotherapy in urothelial cancer 11. Genomic alterations in DNA response and repair-associated genes predicted responses and clinical benefits after cisplatin-based chemotherapy for bladder cancer. Low ATM expression levels were associated with poor overall 5-year survival in patients with gastric cancer undergoing curative surgical resection 12 and in patients with advanced gastric cancer undergoing palliative 1^st^ line XELOX therapy 13. Recently, the phase III GOLD trial failed to show survival benefits in gastric cancer after first-line chemotherapy with olaparib 14.

Accordingly, it may be hypothesized that DDR-related protein defects are associated with poor survival in gastrectomized patients with stage II and III gastric cancer. Therefore, we investigated the relationship between the expression of DDR and gastric cancer patient survival to determine the survival-associated prognostic potential of DDR-related proteins.

## Materials and Methods

### Patients

A total of 217 patients with stage II and III primary gastric cancer were enrolled in this study, who had undergone D2 radical gastrectomy at the Chung-Ang University Hospital, between January 2005 and December 2017. The diagnosis of gastric cancer was confirmed by pathological staining. The cancer staging was performed according to the 7^th^ edition of the American Joint Committee on Cancer 15. Patients with distant metastasis, such as liver metastasis, or peritoneal seeding were excluded. Adjuvant chemotherapy was the standard treatment for gastrectomized patients with stage II and III gastric cancer (unless the patient refused to undergo chemotherapy). This study was approved by the Institutional Review Board of Chung-Ang University Hospital (IRB number: 1981-005-382).

### Immunohistochemistry

The immunohistochemical assessment of MLH1, MSH2, ARID1A, PARP-1, BRCA1, and ATM was performed using formalin-fixed paraffin-embedded (FFPE) tissue samples (Fig. S1).

The mismatch repair proteins MLH1 and MSH2 were scored based on the following threshold: positive when staining was detected in 10% or more of the tumor cell nuclei; negative when staining was detected in less than 10% of the tumor cell nuclei 16.

The PARP-1 staining was scored based on the staining intensity as follows: 0 (negative), 1 (weak), 2 (moderate), and 3 (strong). The percentage of staining distribution of each marker within the tumor cells was recorded. A histochemical (H) score was then calculated as follows: (1 percentage weak), (2 percentage moderate), and (3 percentage strong). The H-score is representative of the overall staining intensity ranges from 0 to 300 17. The PARP-1 staining was scored as follows: positive or high expression, staining achieving H-scores of more than 175; negative or low expression, staining achieving H-scores of less than 175.

The ARID1A staining was scored as follows: negative, undetectable; positive, no loss and focal loss 18. The BRCA1 staining was scored as follows: negative, staining in less than 5% of the tumor cell nuclei; positive, staining in more than 5% of the tumor cell nuclei 19. The ATM assay was evaluated based on the nuclear signal, with the percentage of weakly stained cells over a range of 0-300. A dichotomous classification system was devised whereby the cases were classified as follows: negative, intensity staining in ≤ 10% of the cancer cells (H-score ≤ 10) 20; positive, staining in more than 10% of the cancer cells.

### Assessment

Clinicopathological data, including patient age, sex, tumor-node-metastasis (TNM) stage, lymphatic invasion, venous invasion, perineural invasion, type of surgery, adjuvant chemotherapy, and chemotherapy regimen, were obtained retrospectively from medical records. The clinical outcomes included overall and disease-free survival (OS and DFS, respectively). OS was defined as the period between the gastrectomy and the time of death from any cause. DFS was defined as the period between the gastrectomy and the time of the recurrence of gastric cancer, distant metastasis, diagnosis of another cancer, or death from any cause.

### Statistical analyses

Hazard ratios (HRs) and their corresponding 95% confidence intervals (CI) were stratified using a Cox proportional hazards regression model. Multivariate Cox regression models were constructed for testing significant variables based on the following criterion: P-value < 0.1 (for univariate analysis). The level of statistical significance was defined at P < 0.05. The Kaplan-Meier method was used to estimate the OS and DFS. All statistical analyses were performed using the Statistical Package for Social Sciences (SPSS) version 26.0 (IBM Corp., Armonk, NY, USA).

## Results

### Patients

The baseline characteristics of the patients are shown in Table 1. The median age was 67 years (ranging between 30-90) and 151 participants (69.6%), among the 217 patients studied in total, were men. The histological differentiation of the different cancer types was performed in the 217 patients. The depth of tumor invasion was evaluated as follows: 5.5% as T1 (n = 12, the tumor invades the mucosa or submucosa), 9.7% as T2 (n = 21, the tumor invades the muscularis propria), 47.0% as T3 (n = 102, the tumor invades the subserosal connective tissue without invading the visceral peritoneum or the adjacent structures), and 37.8% as T4 (n = 82, the tumor invades the serosa or the adjacent organs and structures). Lymph node metastasis was detected in 171 patients (78.8%), and 184 gastrectomized patients (84.8%) received adjuvant chemotherapy.

### Expression of DDR-related proteins

**Figure 1:**
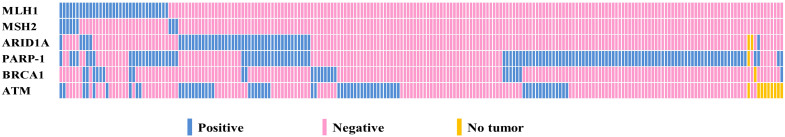
Relationship between the expression levels of six DDR biomarkers (MLH1, MSH2, ARID1A, PARP-1, BRCA1, and ATM) (n = 217).

Fig. 1 shows the relationship between the protein expression levels of MLH1, MSH2, ARID1A, PARP-1, BRCA1, and ATM and occurrence of gastric cancer. The most commonly mutated DDR expression was high PARP-1 (n = 120, 55.3%), followed by ATM (n = 62, 28.6%), ARID1A (n = 45, 20.7%), MLH1 (n = 33, 15.2%), BRCA1 (n = 25, 11.5%), and MSH2 (n = 9, 4.1%). Low PARP-1 expression levels did not depend on the following factors: an age of 65 years or older (P = 0.443) and sex (P = 0.692).

### Association of PARP-1 and other DDR-related protein expressions with survival

The cutoff time for the analyses was January 2020, resulting in a median follow-up of 69.0 months (95% CI = 63.7-74.2 months) including the death of 95 patients (43.8%). The median OS and DFS were 89.0 months (95% CI = 81.1-100.3 months) and 60.0 months (95% CI = 25.0-94.9 months), respectively. One hundred and eight patients (49.8%) relapsed or died during the follow-up period. We evaluated the association between the expression of other DDR-related s MLH1, MSH2, ARID1A, BRCA1, and ATM and survival but observed no statistically significant difference.

**Figure 2:**
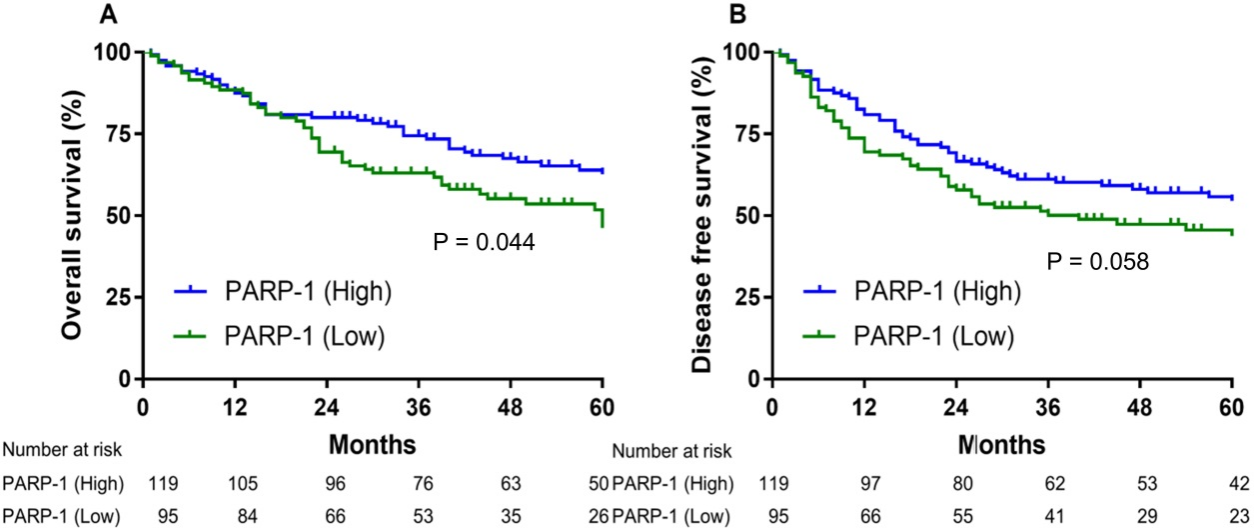
Low PARP-1 expression levels were associated with significantly shorter overall survival in patients with stage II and III gastric cancer **(A)**. The disease-free survival was differentiated on the basis of PARP-1 expression **(B)**.

The low-expression PARP-1 group exhibited a significantly shorter 5-year OS rate than the high-expression PARP-1 group (48.1% vs. 62.7%; HR 1.519, 95% CI = 1.011-2.283, P = 0.044). (Fig. 2A). Although these differences were not statistically significant, the low PARP-1 expression levels were marginally associated with a shorter median DFS, compared to the high PARP-1 expression levels (36.0 months vs. 96.0 months, HR 1.443, 95% CI = 0.998-2.109, P = 0.058) (Fig. 2B).

The univariate OS analysis of the potential prognostic impact of the clinicopathological parameters identified TNM stage, age, sex, lymph node metastasis, lymphatic invasion, perineural invasion, venous invasion, PARP-1, and adjuvant chemotherapy as significant predictors of OS (Table 2). In the multivariate OS analysis, TNM stage (II vs. III) (HR = 5.172, P < 0.001), low PARP-1 expression level (HR = 1.697, P = 0.013), and adjuvant chemotherapy (HR = 0.382, P < 0.001) were the only significant prognostic factors.

The univariate DFS analysis of the potential prognostic impact of the clinicopathological parameters identified TNM stage, age, sex, lymph node metastasis, lymphatic invasion, perineural invasion, venous invasion, and adjuvant chemotherapy as significant predictors of DFS (Table 3). In the multivariate DFS analysis, TNM stage (HR = 3.881, P < 0.001), low PARP-1 expression (HR = 1.547, P = 0.026), and adjuvant chemotherapy (HR = 0.596, P = 0.032) were found to be the only significantly prognostic factors.

### Prognostic value of PARP-1 expression based on adjuvant chemotherapy

**Figure 3:**
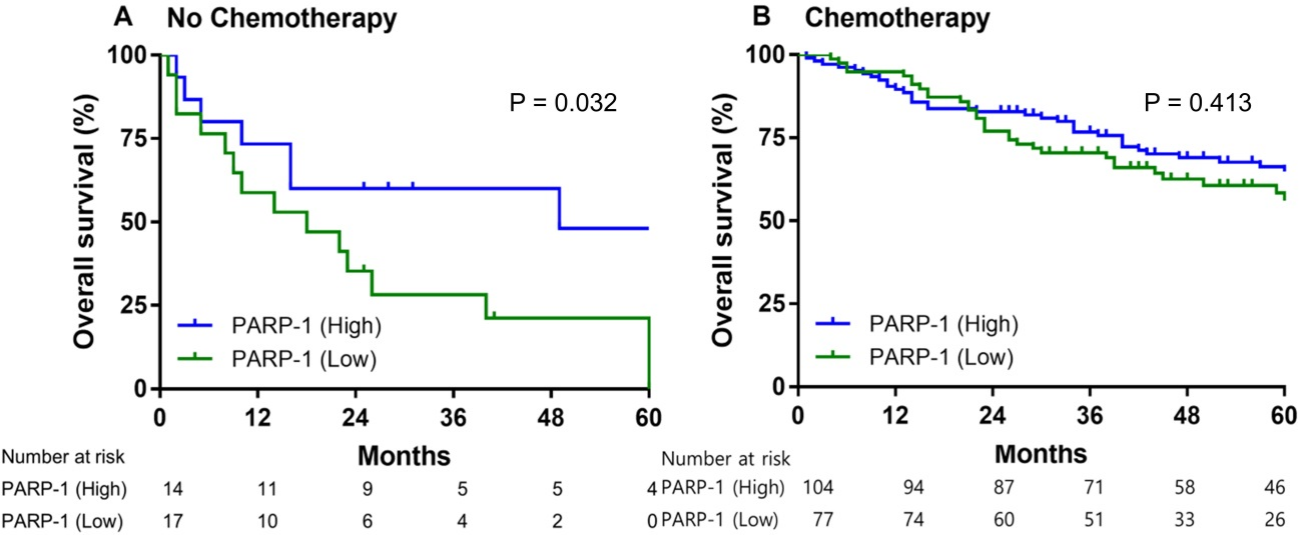
The overall survival of patients with stage II and III gastric cancer exhibiting high compared with those exhibiting low PARP-1 expression levels. Patients not having received **(A)** and having received adjuvant chemotherapy **(B)**.

Of the patients with stage II and III gastric cancer who did not receive adjuvant chemotherapy, the low-expression PARP-1 group had significantly shorter median overall survival than the high-expression PARP-1 group (14.0 months vs. 49.0 months, HR = 2.659, 95% CI = 1.085-6.517, P = 0.032) (Fig. 3A). However, the 5-year overall survival rate in patients who had received adjuvant chemotherapy did not significantly differ between the low- and high-PARP-1 expression groups (59.2% vs. 64.9%, HR = 1.217, 95% CI = 0.760-1.949, P = 0.413) (Fig. 3B).

### Impact of PARP-1 expression and other DDR-related protein expressions on adjuvant chemotherapy regimen

**Figure 4:**
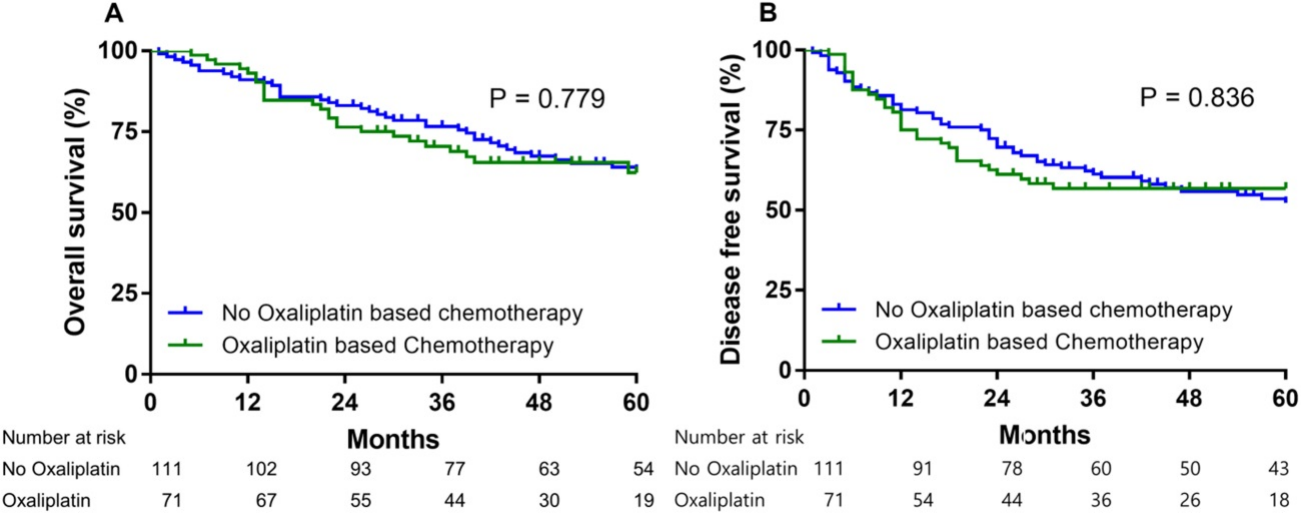
Overall survival (A) and disease free survival (B) according to oxaliplatin based adjuvant chemotherapy.

**Figure 5:**
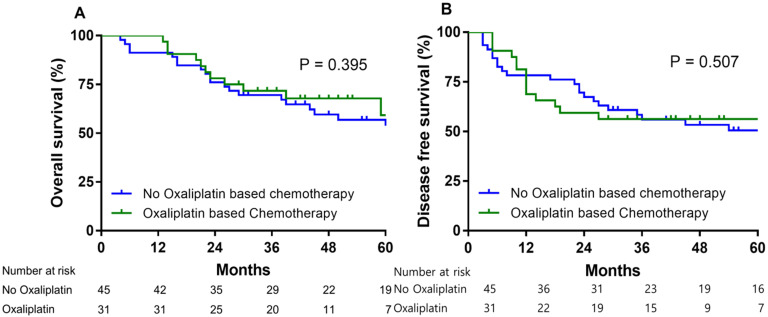
Overall survival (A) and disease free survival (B) according to oxaliplatin based adjuvant chemotherapy vs. no oxaliplatin based chemotherapy in low PARP-1 expression gastric cancer group.

184 patients received adjuvant chemotherapy after gastrectomy. For 72 patients who received adjuvant oxaliplatin-based chemotherapy for resectable gastric cancer. The 5 year OS rate and 5 year DFS rate with adjuvant based chemotherapy for these 72 patients were 62.3% and 56.8%, respectively (Fig. 4). In low PARP-1 expression group, oxaliplatin based adjuvant chemotherapy group had not significantly different 5 year OS rate than no oxaliplatin based adjuvant chemotherapy group (63.9% vs. 54.9%, HR = 0.716, 95% CI= 0.331-1.547, P = 0.395) (Fig. 5A). In low PARP-1 expression group, oxaliplatin based adjuvant chemotherapy group had not significantly different DFS than no oxaliplatin based adjuvant chemotherapy group (74 months vs. 45 months, HR = 0.788, 95% CI= 0.389-1.594, P = 0.507) (Fig. 5B).

In low BRCA expression group, oxaliplatin based adjuvant chemotherapy group had not significantly different median 5 year OS than no oxaliplatin based adjuvant chemotherapy group (74.0 months vs. 43.0 months, HR = 0.993, 95% CI= 0.296-3.333, P = 0.992).

In low ATM expression group, oxaliplatin based adjuvant chemotherapy group had not significantly different 5 year OS rate than no oxaliplatin based adjuvant chemotherapy group (40.0% vs. 72.0%, HR = 2.414, 95% CI= 0.987-5.907, P = 0.054).

## Discussion

This study presented the results of the immunohistochemical assessment of the expression of DDR-protein in 271 patients with stage II and III gastric cancer. The results showed that low PARP-1 expression levels were associated with poor prognosis when gastrectomized patients underwent lymph node dissection. The low-expression PARP-1 group had significantly shorter median OS than the high-expression PARP-1 group in the case of patients with gastric cancer who did not receive adjuvant chemotherapy. However, the 5-year OS in patients who had received adjuvant chemotherapy did not significantly differ between the low- and high-expression PARP-1 groups.

In low expression PARP-1 patients, the prognosis of patients who received oxaliplatin based adjuvant chemotherapy was similar to the prognosis of patients who received no oxaplatin based adjuvant chemotherapy group. It suggested that the oxalipatin based adjuvant chemotherapy may not affect survival according to the low of PARP-1 expression. This study presented the clinical implication of DDR gene, but it should not find a correlation with clinical outcomes and other genes of DDR gene except of PARP-1 gene.

As reported in a previous study, the incidence of low PARP-1 expression was 47.2% in gastric cancer 21; the incidence of low PARP-1 expression reported herein (44.7%) was similar to this value. Another study reported that the incidences of the loss of ATM, BRCA1, and ARID1A expression were 18-22 13, 14, 17.5 22, and 11-21% in gastric cancer 23, respectively. The expression of other DDRs were similar in our study. This study presented the clinical implications of the expression of DDR-related genes but did not highlight any correlation between clinical outcomes and the expression of other DDR-related genes (except PARP-1).

The PARP protein family comprises 17 enzymes involved in the regulation of the cell cycle, genome stability, transcription 24, DNA damage response 25, and cell death. High PARP-1 expression is associated with higher pathologically complete remission rates after neoadjuvant chemotherapy in breast cancer 26. Inhibition of PARP-1 expression improves the efficacy of chemotherapy by impairing DNA repair 27. These results provide the rationale behind the attempts to supplement chemotherapy with a PARP inhibitor in the presence of high PARP-1 expression levels.

PARP-1 expression is reportedly associated with a good prognosis in other cancer types, including breast cancer 28 and non-small-cell lung cancer 29. Aiad et al 28 demonstrated that high PARP-1 expression levels were significantly associated with improved OS in locally advanced breast cancer. Klauschen et al 29 described that low PARP-1 expression levels were associated with a poor prognosis in pancreatic cancer. However, Liu et al 21 demonstrated that high PARP-1 expression levels were associated with significantly reduced DFS and OS in patients with gastric cancer. These studies indicate that PARP-1 expression could play different roles at different stages of tumors and treatments. In our study, low PARP-1 expression levels were associated with significantly poor DFS and OS in gastrectomized patients with stage II and stage III gastric cancer. According to our multivariate analysis, not only low PARP-1 expression levels, but also the TNM stage and adjuvant chemotherapy, were independent prognostic factors in gastric cancer.

Adjuvant chemotherapy after surgery in gastric cancer is a standard current treatment for stage II and III gastric cancer 3. Patients who had received adjuvant chemotherapy showed similar OS rates irrespective of the PARP-1 expression levels. The prognostic effect was significant in the TNM stage and upon adjuvant chemotherapeutic treatment. Based on these observations, low PARP-1 expression levels may improve the efficacy of adjuvant chemotherapy when treating gastric cancer. Low PARP-1 expression levels could potentially favor the development of mutations through dysfunctional DNA repair, and PARP-1 could enhance the chemotherapeutic benefits with regard to survival. In gastric cancer, the high expression of PARP-1 may lead to the suppression of the activities of NAD+ and ATP, which in turn, may cause cell death 30.

The cytotoxic effects of platinum, including oxaliplatin, are to trigger a variety of downstream signaling pathways. High PARP-1 expression maybe affinity to the most common 1,2-d(GpG) and this affinity decreases upon automodification which implicates the role of PARP-1 in repair of platinum-induced DNA damage. Our data suggest that the low PARP-1 expression may have a role as predictive biomarkers for the response to adjuvant chemotherapy. However, the survival of oxalipatin based adjuvant chemotherapy was not better than the survival of no oxaliplatin based chemotherapy in patients with gastric cancer stage II or III. The 3 year DFS rate as 56% in our study with oxaliplatin base adjuvant chemotherapy was shorter than the 3 year DFS rate as 78% in the classic study^4^. In our study, the oxalipatin base adjuvant chemotherapy group was significantly more included stage III (P = 0.008), lymph node positive (P = 0.038), lymphatic invasion (P <0.001) and venous invasion (P = 0.005). The OS and DFS in the oxaliplatin base adjuvant chemotherapy group was worse because the factors that are not good for survival were included.

In conclusion, the present study demonstrated that low PARP-1 expression levels are associated with poor overall survival and disease-free survival. Low PARP-1 expression levels could be an indicator of poor prognosis, particularly in gastrectomized patients with stage II and III gastric cancer. Patients with stage II and stage III gastric cancer and low PARP-1 expression levels benefited from adjuvant chemotherapy. Therefore, adjuvant chemotherapy is required in patients with gastric cancer who display low PARP-1 expression levels.

## Supplementary Material

Supplementary figure.

## Figures and Tables

**Table 1:** Baseline characteristics.

Characteristics	Total (n = 217)
Age - years	
median	67
range	30-90
Age > 65	121 (55.8%)
Sex, n (%)	
Male	151 (69.6%)
Female	66 (30.4%)
Histological differentiation	
Well differentiation	4 (1.8%)
Moderated differentiation	74 (34.1%)
High differentiation	116 (53.5%)
Signet ring cell	18 (8.3%)
Other	5 (2.3%)
Invasion depth	
T1	12 (5.5%)
T2	21 (9.7%)
T3	102 (47.0%)
T4	82 (37.8%)
Lymph node metastasis	
Negative	46 (21.2%)
Positive	171 (78.8%)
Lymphatic invasion	
Negative	71 (32.7%)
Positive	146 (67.3%)
Venous invasion	
Negative	78 (35.9%)
Positive	139 (64.1%)
Perineural invasion	
Negative	88 (40.6%)
Positive	129 (59.4%)
TNM stage	
II	98 (45.2%)
III	119 (54.8%)
Surgery	
Total gastrectomy	75 (34.6%)
Subtotal gastrectomy	142 (65.4%)
Adjuvant chemotherapy	
No	33 (15.2%)
Yes	184 (84.8%)
Adjuvant chemotherapy regimen	N=184(100%)
FL	25 (13.6%)
S-1	84 (45.7%)
XELOX	57 (31.0%)
FOLFOX	4 (2.2%)
Other	14 (7.6%)

TNM: tumor-node-metastasis; FL: 5-fluorouracil and leucovorin; XELOX: capecitabine and oxaliplatin; FOLFOX: 5-fluorouracil, leucovorin, and oxaliplatin.

**Table 2:** Univariate and Multivariate Cox regression models for the analysis of factors affecting overall survival.

		Univariate Cox		Multivariate Cox	
		Regression model		Regression model	
		HR (95% CI)	P-value	HR (95% CI)	P-value
**Stage**	**II vs. III**	4.858	< 0.001	5.172	< 0.001
		(2.929-8.055)		(2.608-10.256)	
**Age**	**< 65 vs. ≥ 65**	1.606	0.027	1.503	0.070
		(1.054-2.448)		(0.968-2.336)	
**Sex**	**Male vs. Female**	1.226	0.349		
		(0.800-1.879)			
**Lymph node**	**No vs. Yes**	3.143	0.001	1.069	0.883
		(1.580-6.251)		(0.437-2.615)	
**Lymphatic invasion**	**No vs. Yes**	1.905	0.008	0.582	0.332
		(1.180-3.074)		(0.195-1.738)	
**Perineural invasion**	**No vs. Yes**	1.769	0.012	0.922	0.752
		(1.131-2.767)		(0.559-1.522)	
**Venous invasion**	**No vs. Yes**	1.903	0.007	1.519	0.428
		(1.196-3.027)		(0.540-4.271)	
**MLH1**	**High vs. Low**	0.976	0.930		
		(0.560-1.699)			
**MSH2**	**High vs. Low**	0.599	0.384		
		(0.189-1.898)			
**ARID1A**	**High vs. Low**	1.041	0.871		
		(0.640-1.693)			
**PARP-1**	**High vs. Low**	1.519	0.044	1.697	0.013
		(1.011-2.283)		(1.120-2.573)	
**BRCA1**	**High vs. Low**	1.312	0.364		
		(0.730-2.359)			
**ATM**	**High vs. Low**	0.946	0.809		
		(0.605-1.480)			
**Adjuvant**	**No vs. Yes**	0.323	<0.001	0.382	<0.001
**Chemotherapy**		(0.201-0.520)		(0.233-0.625)	

ARID1A: AT-rich interaction domain 1; PARP-1: Poly adenosine diphosphate-ribose polymerase 1; BRCA1: Breast cancer susceptibility gene 1; ATM: ataxia-telangiectasia mutated.

**Table 3:** Univariate and Multivariate Cox regression models for the analysis of factors affecting disease-free survival.

		Univariate Cox		Multivariate Cox	
		Regression model		Regression model	
		HR (95% CI)	P-value	HR (95% CI)	P-value
**Stage**	**II vs. III**	4.047	< 0.001	3.881	< 0.001
		(2.583-6.342)		(2.109-7.142)	
**Age**	**< 65 vs. ≥ 65**	1.572	0.024	1.489	0.054
		(1.063-2.324)		(0.994-2.232)	
**Sex**	**Male vs. Female**	1.163	0.460		
		(0.779-1.735)			
**Lymph node**	**No vs. Yes**	2.410	0.003	0.864	0.716
		(1.350-4.305)		(0.393-1.900)	
**Lymphatic invasion**	**No vs. Yes**	1.932	0.004	0.666	0.408
		(1.235-3.024)		(0.254-1.744)	
**Perineural invasion**	**No vs. Yes**	2.023	0.001	1.245	0.352
		(1.327-3.085)		(0.785-1.974)	
**Venous invasion**	**No vs. Yes**	2.017	0.002	1.535	0.348
		(1.304-3.120)		(0.627-3.756)	
**MLH1**	**High vs. Low**	0.867	0.609		
		(0.501-1.499)			
**MSH2**	**High vs. Low**	0.556 (0.176-1.758)	0.317		
**ARID1A**	**High vs. Low**	1.059	0.805		
		(0.672-1.668)			
**PARP-1**	**High vs. Low**	1.443	0.058	1.547	0.026
		(0.988-2.109)		(1.054-2.270)	
**BRCA1**	**High vs. Low**	1.279	0.377		
		(0.741-2.208)			
**ATM**	**High vs. Low**	1.065	0.768		
		(0.768-1.065)			
**Adjuvant**	**No vs. Yes**	0.452	0.001	0.596	0.032
**Chemotherapy**		(0.285-0.718)		(0.371-0.957)	

ARID1A: AT-rich interaction domain 1; PARP-1: Poly adenosine diphosphate-ribose polymerase 1; BRCA1: Breast cancer susceptibility gene 1; ATM: ataxia-telangiectasia mutated.

## Abbreviations

DDR: DNA damage response
DFS: disease-free survival
OS: overall survival
ARID1A: at-rich interaction domain 1
PARP-1: poly adenosine diphosphate-ribose polymerase 1
BRAC1: breast cancer susceptibility gene 1
ATM: ataxia-telangiectasia mutated
FFPE: formalin-fixed paraffin-embedded
HR: hazard ratio
CI: confidence interval

## References

[1] Bray F, Ferlay J, Soerjomataram I, Siegel RL, Torre LA, Jemal A (2018). Global cancer statistics 2018: GLOBOCAN estimates of incidence and mortality worldwide for 36 cancers in 185 countries. CA Cancer J Clin.

[2] Muneoka Y, Akazawa K, Ishikawa T, Ichikawa H, Nashimoto A, Yabusaki H (2016). Nomogram for 5-year relapse-free survival of a patient with advanced gastric cancer after surgery. Int J Surg.

[3] Noh SH, Park SR, Yang HK, Chung HC, Chung IJ, Kim SW (2014). Adjuvant capecitabine plus oxaliplatin for gastric cancer after D2 gastrectomy (CLASSIC): 5-year follow-up of an open-label, randomised phase 3 trial. Lancet Oncol.

[4] Bang YJ, Kim YW, Yang HK, Chung HC, Park YK, Lee KH (2012). Adjuvant capecitabine and oxaliplatin for gastric cancer after D2 gastrectomy (CLASSIC): a phase 3 open-label, randomised controlled trial. Lancet.

[5] Hartwell LH, Weinert TA (1989). Checkpoints: controls that ensure the order of cell cycle events. Science.

[6] Kastan MB, Bartek J (2004). Cell-cycle checkpoints and cancer. Nature.

[7] Felsberg J, Thon N, Eigenbrod S, Hentschel B, Sabel MC, Westphal M (2011). Promoter methylation and expression of MGMT and the DNA mismatch repair genes MLH1, MSH2, MSH6 and PMS2 in paired primary and recurrent glioblastomas. Int J Cancer.

[8] Shen J, Peng Y, Wei L, Zhang W, Yang L, Lan L (2015). ARID1A Deficiency Impairs the DNA Damage Checkpoint and Sensitizes Cells to PARP Inhibitors. Cancer Discov.

[9] Durkacz BW, Omidiji O, Gray DA, Shall S (1980). (ADP-ribose)n participates in DNA excision repair. Nature.

[10] Savage KI, Gorski JJ, Barros EM, Irwin GW, Manti L, Powell AJ (2014). Identification of a BRCA1-mRNA splicing complex required for efficient DNA repair and maintenance of genomic stability. Mol Cell.

[11] Miron B, Hoffman-Censits JH, Anari F, O'Neill J, Geynisman DM, Zibelman MR (2020). Defects in DNA Repair Genes Confer Improved Long-term Survival after Cisplatin-based Neoadjuvant Chemotherapy for Muscle-invasive Bladder Cancer. Eur Urol Oncol.

[12] Kim JW, Im SA, Kim MA, Cho HJ, Lee DW, Lee KH (2014). Ataxia-telangiectasia-mutated protein expression with microsatellite instability in gastric cancer as prognostic marker. Int J Cancer.

[13] Klempner SJ, Bhangoo MS, Luu HY, Kim ST, Chao J, Kim KM (2018). Low ATM expression and progression-free and overall survival in advanced gastric cancer patients treated with first-line XELOX chemotherapy. J Gastrointest Oncol.

[14] Bang YJ, Xu RH, Chin K, Lee KW, Park SH, Rha SY (2017). Olaparib in combination with paclitaxel in patients with advanced gastric cancer who have progressed following first-line therapy (GOLD): a double-blind, randomised, placebo-controlled, phase 3 trial. Lancet Oncol.

[15] Sobin LH, Compton CC (2010). TNM seventh edition: what's new, what's changed: communication from the International Union Against Cancer and the American Joint Committee on Cancer. Cancer.

[16] Sarode VR, Robinson L (2019). Screening for Lynch Syndrome by Immunohistochemistry of Mismatch Repair Proteins: Significance of Indeterminate Result and Correlation With Mutational Studies. Arch Pathol Lab Med.

[17] Green AR, Caracappa D, Benhasouna AA, Alshareeda A, Nolan CC, Macmillan RD (2015). Biological and clinical significance of PARP1 protein expression in breast cancer. Breast Cancer Res Treat.

[18] Allo G, Bernardini MQ, Wu RC, Shih Ie M, Kalloger S, Pollett A (2014). ARID1A loss correlates with mismatch repair deficiency and intact p53 expression in high-grade endometrial carcinomas. Mod Pathol.

[19] Garg K, Levine DA, Olvera N, Dao F, Bisogna M, Secord AA (2013). BRCA1 immunohistochemistry in a molecularly characterized cohort of ovarian high-grade serous carcinomas. Am J Surg Pathol.

[20] Miller RM, Nworu C, McKee L, Balcerzak D, Pham L, Pugh J (2020). Development of an Immunohistochemical Assay to Detect the Ataxia-Telangiectasia Mutated (ATM) Protein in Gastric Carcinoma. Appl Immunohistochem Mol Morphol.

[21] Liu Y, Zhang Y, Zhao Y, Gao D, Xing J, Liu H (2016). High PARP-1 expression is associated with tumor invasion and poor prognosis in gastric cancer. Oncol Lett.

[22] Kim HS, Hwang IG, Min HY, Bang YJ, Kim WH (2019). Clinical significance of BRCA1 and BRCA2 mRNA and protein expression in patients with sporadic gastric cancer. Oncol Lett.

[23] Inada R, Sekine S, Taniguchi H, Tsuda H, Katai H, Fujiwara T (2015). ARID1A expression in gastric adenocarcinoma: clinicopathological significance and correlation with DNA mismatch repair status. World J Gastroenterol.

[24] Kim MY, Mauro S, Gevry N, Lis JT, Kraus WL (2004). NAD+-dependent modulation of chromatin structure and transcription by nucleosome binding properties of PARP-1. Cell.

[25] Masaoka A, Horton JK, Beard WA, Wilson SH (2009). DNA polymerase beta and PARP activities in base excision repair in living cells. DNA Repair (Amst).

[26] von Minckwitz G, Muller BM, Loibl S, Budczies J, Hanusch C, Darb-Esfahani S (2011). Cytoplasmic poly(adenosine diphosphate-ribose) polymerase expression is predictive and prognostic in patients with breast cancer treated with neoadjuvant chemotherapy. J Clin Oncol.

[27] Ashworth A (2008). A synthetic lethal therapeutic approach: poly(ADP) ribose polymerase inhibitors for the treatment of cancers deficient in DNA double-strand break repair. J Clin Oncol.

[28] Aiad HA, Kandil MA, El-Tahmody MA, Abulkheir IL, Abulkasem FM, Elmansori AA (2015). The prognostic and predictive significance of PARP-1 in locally advanced breast cancer of Egyptian patients receiving neoadjuvant chemotherapy. Appl Immunohistochem Mol Morphol.

[29] Klauschen F, von Winterfeld M, Stenzinger A, Sinn BV, Budczies J, Kamphues C (2012). High nuclear poly-(ADP-ribose)-polymerase expression is prognostic of improved survival in pancreatic cancer. Histopathology.

[30] Carson DA, Carrera CJ, Wasson DB, Yamanaka H (1988). Programmed cell death and adenine deoxynucleotide metabolism in human lymphocytes. Adv Enzyme Regul.

