# Supplementary material for: Low PARP-1 expression level is an indicator of poor prognosis in patients with stage II and III gastric cancer
Source: J Cancer. 2022 Jan 1;13(3):869–76. doi: 10.7150/jca.65145 (PMC8824884; doi:10.7150/jca.65145)

**Fig S1.** Expression of DNA damage response (DDR) in gastric cancer. Immunohistochemical staining for MLH1 (A and B), MSH2 (C and D), ARID1A (E and F), PARP-1 (G and H), BRCA1 (I and J), and ATM (K and L). Low expression of MLH1 (A), MSH2 (C), ARID1A (E), PARP-1 (G), BRCA1 (I), and ATM (K). Magnification: 200 ×

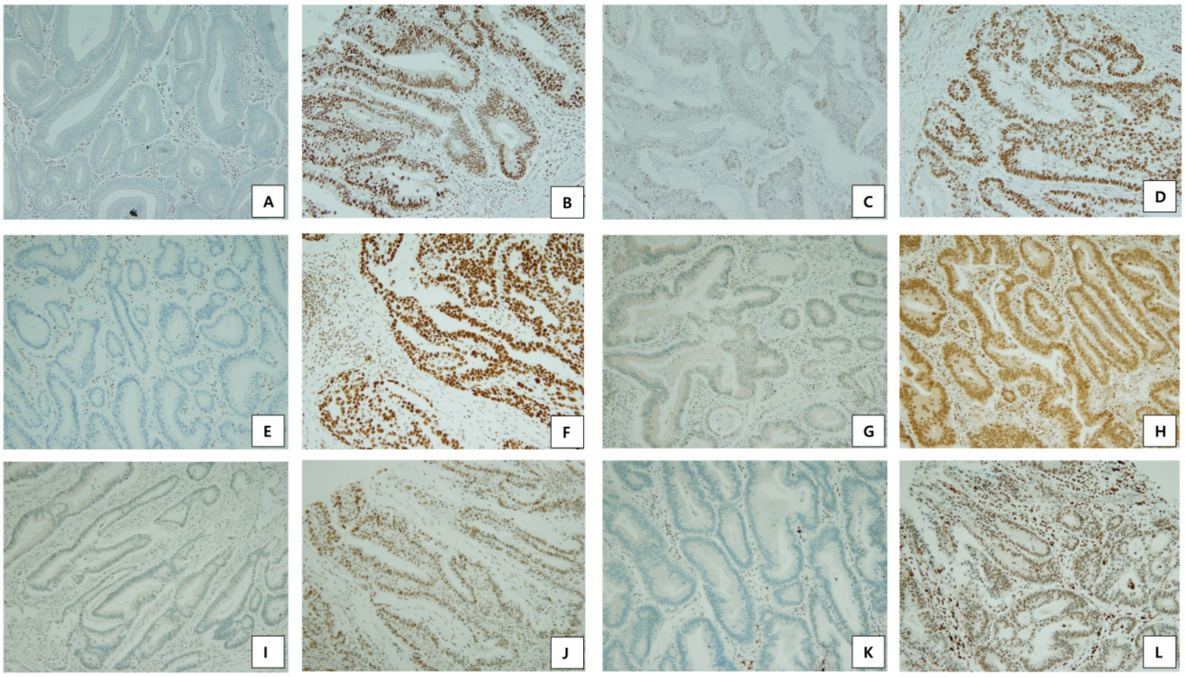

Supplement: Supplementary file 1 — Supplementary figure. [file jcav13p0869s1.pdf]
